# Post-operative radiotherapy is beneficial for T1/T2 triple negative breast cancer patients with four or more positive lymph nodes

**DOI:** 10.18632/oncotarget.17170

**Published:** 2017-04-18

**Authors:** Lin Chen, Jinfeng Zhang, Jiayi Chen, Lili Liu, Lili Liang, Zhiyi Shangguan, Dandan Wang

**Affiliations:** ^1^ Department of Radiation Therapy Technology Center, Harbin Medical University Cancer Hospital, Harbin, China; ^2^ Department of Breast Surgery, Harbin Medical University Cancer Hospital, Harbin, China; ^3^ Department of Radiological Physics, Harbin Medical University Cancer Hospital, Harbin, China

**Keywords:** triple negative breast cancer, adjuvant radiotherapy, positive nodes, survival

## Abstract

The efficacy of adjuvant radiotherapy for the treatment of triple negative breast cancer patients with varying numbers of positive lymph nodes is not clear. We assessed the association between adjuvant radiotherapy and survival in 943 T1/T2 triple negative breast cancer patients treated at our institute between 2008 and 2012. We determined that post-operative radiotherapy improved overall survival (OS), disease-free survival (DFS), and local recurrence-free survival (LRFS) in patients with ≥ 4 positive nodes (*p* = 0.037, *p* = 0.035, and *p* = 0.012, respectively). Although Cox regression analysis demonstrated that radiotherapy was a significant prognostic factor in triple negative breast cancer with ≥ 4 positive nodes, post-operative radiotherapy had no clear effect on OS, DFS, or LRFS in patients with 1-3 positive nodes (*p* = 0.849, *p* = 0.860, and *p* = 0.162, respectively). The prognosis (i.e., OS, DFS, and LRFS) of triple negative breast cancer patients without lymph node metastasis who underwent breast-conserving surgery and post-operative radiotherapy was similar to that of patients who underwent mastectomy alone (*p* = 0.336, *p* = 0.537, and *p* = 0.978, respectively). Our findings demonstrate that post-operative radiotherapy is beneficial for T1/T2 triple negative breast cancer patients with ≥ 4 positive lymph nodes.

## INTRODUCTION

Breast cancer is the most common malignant tumor that occurs in women worldwide and it is a leading cause of cancer-related deaths. There were an estimated 231,840 new cases in the United States in 2015 [1]. Breast cancer is classified based on three molecular markers: the estrogen receptor (ER), progesterone receptor (PR), and human epidermal growth factor receptor 2 (HER2). It is further classified into distinct subtypes: luminal A, luminal B, HER2, and triple negative [2–6]. Triple negative breast cancer (ER-, PR-, and HER2-negative) is associated with an increased risk of early metastasis and local recurrence relative to the other breast cancer subtypes [7]. Chemotherapy is the primary method of treatment of triple negative breast cancer patients, and the potential benefits of adjuvant radiotherapy are not yet clear [8]. Treatment of breast cancer patients is therefore challenging given the lack of treatment options.

Several studies have shown that radiotherapy can reduce the rate of loco-regional recurrence and prolong survival in breast cancer patients [9, 10]. Radiotherapy reduced the overall death rate at 15 years by 3.8% (25.2% to 21.4%) in patients who underwent breast-conserving surgery [11]. Adjuvant radiotherapy following breast-conserving surgery was more effective than mastectomy alone [12]. However, the effectiveness of post-mastectomy radiotherapy in patients with 1–3 positive lymph nodes is still controversial. Some studies have shown that post-mastectomy radiotherapy is beneficial for patients with 1–3 positive nodes [10, 13–15]. However, McBride et al. reported that patients with 1–3 positive nodes without risk features had a low rate of loco-regional recurrence, even without radiotherapy [16]. The 13^th^ St. Gallen International Breast Cancer Conference (2013) Expert Panel supported less extensive axillary treatment and shorter durations of radiation therapy for early breast cancer patients [17].

Post-mastectomy radiotherapy in patients with stage I–II triple negative breast cancer was associated with a 13.7% reduction in loco-regional recurrence and an 11.7% increase in overall survival (OS) in a large multicenter randomized trial from China [18]. A large retrospective cohort study found that the patients who were treated with breast-conserving surgery and radiotherapy had longer local recurrence-free survival (LRFS) compared to T1–2/N0 triple negative breast cancer patients who underwent mastectomy alone [12]. However, these results were not consistent with those of other studies [19–22]. Adjuvant radiotherapy was less beneficial for triple negative breast cancer patients who underwent breast-conserving surgery and radiotherapy compared to those who underwent mastectomy alone.

Heilongjiang Province is located in the northeast region of China. Breast cancer patients in this region typically undergo mastectomy rather than breast-conserving surgery. Thus, adjuvant radiotherapy following mastectomy could be particularly beneficial to triple negative breast cancer patients in this province. In this study, we evaluated the impact of adjuvant radiotherapy on the survival of triple negative breast cancer patients with 1–3, or ≥ 4 positive lymph nodes and suggest revised criteria for adjuvant radiotherapy in triple negative breast cancer.

## RESULTS

**Figure 1:**
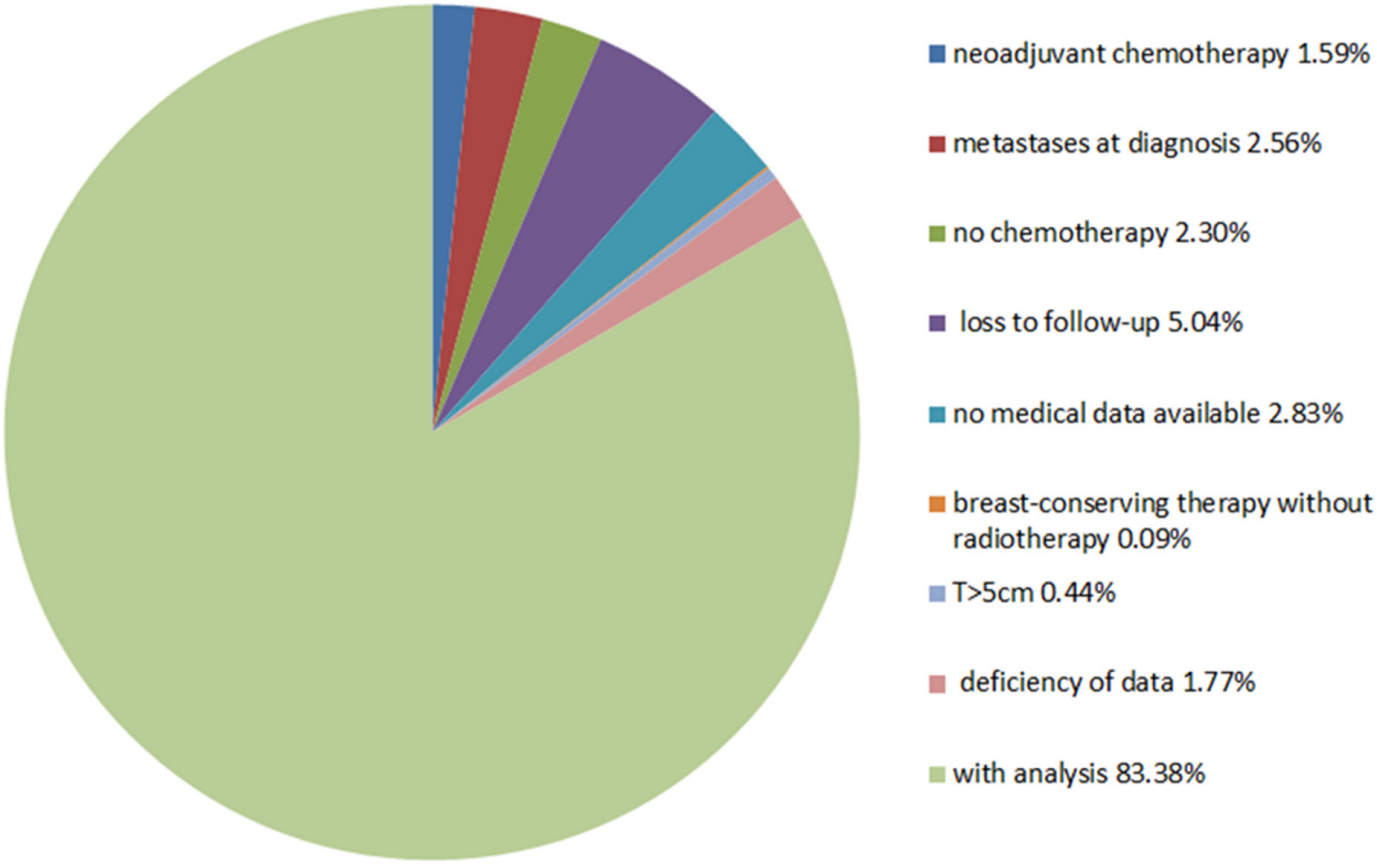
Distribution triple negative breast cancer patients

We identified 1,131 triple negative breast cancer patients (ER-, PR-, and HER2-negative) who were treated at our institute between 2008 and 2012. Patients who presented with metastases at the time of diagnosis (29; 2.56%), not received chemotherapy (26; 2.30%), had a tumor size > 5 cm (5; 0.44%), were lost to follow-up (57; 5.04%), received neo-adjuvant chemotherapy (18; 1.59%), had no medical data available (32; 2.83%), deficiency of data (20:1.77%), or who underwent breast-conserving surgery without radiotherapy (1; 0.09%) were excluded from the study. We analyzed the remaining 943 triple negative breast cancer patients. The distribution of patients is shown in Figure 1.

The characteristics of the patients are shown in Table 1. Of the 943 patients, 295 (31.3%) were ≤ 45 and 648 (68.7%) were > 45 years of age. There were 417 (44.2%) and 526 (55.8%) patients who presented with a tumor size ≤ 2 cm and 2 cm < T ≤ 5 cm, respectively. There were 504 (53.5%) patients who presented with axillary node negative disease, 237 (25.1%) with 1–3 positive lymph nodes, and 202 (21.4%) with ≥ 4 lymph node metastases. There were 256 (27.1%) patients who presented with Ki67 index values < 14% and 687 (72.9%) with values ≥ 14%. Finally, there were 581 (61.6%) P53-positive and 362 (36.4%) P53-negative patients. All patients were treated with surgery and chemotherapy. There were 38 (4.0%) patients who underwent breast-conserving surgery and 905 (96.0%) who underwent mastectomy. We also analyzed the relationship between the number of positive lymph nodes and various clinicopathological features of breast cancer patients. We found that the number of positive lymph nodes was correlated with tumor size (*p* < 0.0001) and histological grade (*p* < 0.0001). The choice of radiotherapy was primarily determined by the number of positive lymph nodes (*p* < 0.0001) (Table 1).

**Table 1:** Characteristics of 943 patients with triple negative breast cancer between 2008 and 2012

Characteristics	Total patients	Lymph node status			*p*-value
		ln0	ln1-3	ln≥4	
	943, n(%)	504(53.5)	237(25.1)	202(21.4)	
Age(years)					
≤45	295(31.3)	149	85	61	0.21
>45	648(68.7)	355	152	141	
Tumor size					
T≤2cm	417(44.2)	299	72	46	<0.0001
2cm<T≤5cm	526(55.8)	205	165	156	
Ki67					
<14%	256(27.1)	132	58	66	0.122
≥14%	687(72.9)	372	179	136	
p53					
positive	581(61.6)	320	136	125	0.279
negative	362(36.4)	184	101	77	
Histological grade					
G1-G2	785(83.2)	448	203	134	<0.0001
G3	158(16.8)	56	34	68	
Post-operative radiotherapy					
Yes	191(20.3)	38	39	114	<0.0001
No	752(79.7)	466	198	88	
Surgery					
Breast-conserving surgery	38(4.0)	38	*	*	—
Mastectomy	905(96.0)	466	237	202	
Chemotherapy					
Yes	943	504	237	202	—
No	*	*	*	*	

*the patients who were not conform to the criteria or too few were excluded.

**Figure 2:**
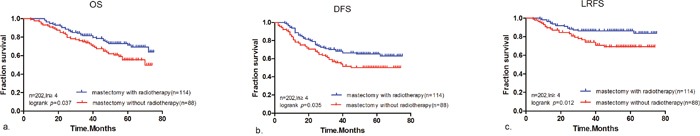
Kaplan-Meier analysis showing **(a)** OS, **(b)** DFS, and **(c)** LRFS for T1/T2 triple negative breast cancer patients with ≥ 4 positive lymph nodes who were treated with mastectomy without radiotherapy (n = 88) or mastectomy with radiotherapy (n = 114).

A total of 191/943 (20.3%) patients underwent post-operative radiotherapy. The median duration of follow-up was 53 months (range 4–78 months). The number of patients who had events (e.g., death, any recurrence, local regional recurrences) in different lymph nodes listed in Table 2. Of the 202 patients with ≥ 4 positive lymph nodes, 114 received post-mastectomy radiotherapy. All patients with ≥ 4 positive lymph nodes underwent mastectomy. Kaplan-Meier survival curves were generated to evaluate the effects of post-mastectomy radiotherapy on OS, DFS, and LRFS. Importantly, post-mastectomy radiotherapy improved OS, DFS, and LRFS in triple negative breast cancer patients (*p =* 0.035, *p* = 0.037, and *p* = 0.012, respectively, Figure 2a, 2b, 2c).

**Table 2:** Patients who had events (Death, Any recurrence, Local regional recurrences) in different lymph nodes

Lymph node status	Total patients	Post-operative radiotherapy	Death	Any recurrence	Local regional recurrences
	943			n(%)	
ln≥4	114	Yes	32(28.07)	40(35.09)	16(14.04)
	88	No	37(42.05)	43(48.86)	24(27.27)
ln1-3	39	Yes	6(15.38)	9(23.07)	3(7.7)
	198	No	32(16.16)	44(22.22)	32(16.16)
ln0	38	Yes	3(7.89)	4(10.52)	1(2.63)
	466	No	61(13.09)	65(13.94)	12(2.58)

Univariate and multivariate survival analyses were performed to evaluate the impact of radiotherapy and clinicopathological factors (e.g., age, tumor size, histological grade, Ki67 index, and P53 status) on the prognosis of triple negative breast cancer patients with ≥ 4 positive lymph nodes. Univariate Cox regression analysis indicated that tumor size (*p* = 0.034) and post-mastectomy radiotherapy (*p* = 0.04) were significant predictors of prognosis. Multivariate Cox regression analysis of OS demonstrated that tumor size (*p* = 0.032) and post-mastectomy radiotherapy (*p* = 0.036) were significant prognostic factors (Table 3). The results of the univariate and multivariate Cox regression analysis of DFS and LRFS were similar to those of OS (Supplementary Table 1 and 2).

**Table 3:** Prognostic factors of OS in triple negative breast cancer patients with ≥ 4 positive lymph nodes using the Cox proportional hazards model

Variables	Hazard Ratio (Univariate 95% CI)	*p*-value	Hazard Ratio (Multivariate 95% CI)	*p*-value
Age (years)				
≥45 vs. <45	1.394(0.797-2.439)	0.244		
Tumor size				
≥2cm vs. <2cm	2.064(1.055-4.037)	0.034	2.087(1.067-4.083)	0.032
Histological grade				
G1-2 vs. G3	0.887(0.492-1.597)	0.689		
Ki67 status				
≥14% vs.< 14%	1.006(0.610-1.660)	0.98		
P53 status				
Positive vs. Negative	1.138(0.699-1.852)	0.603		
Post-mastectomy radiotherapy				
Yes vs. No	0.608(0.379-0.976)	0.04	0.602(0.375-0.967)	0.036

**Figure 3:**
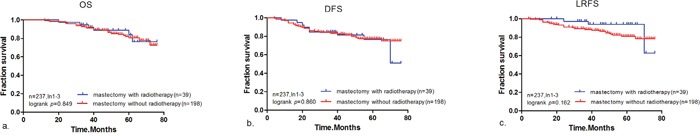
Kaplan-Meier analysis showing **(a)** OS, **(b)** DFS, and **(c)** LRFS for patients with triple negative breast cancer T1/T2 triple negative breast cancer patients with 1−3 positive lymph nodes who were treated with mastectomy without radiotherapy (n = 198) or mastectomy with radiotherapy (n = 39).

Of the 237 patients with 1–3 positive lymph nodes, 39 underwent post-mastectomy radiotherapy. There were few triple negative breast cancer patients with 1–3 positive lymph nodes who underwent breast-conserving surgery. Therefore, all of the patients we selected with 1–3 positive lymph nodes underwent mastectomy. We analyzed the impact of post-mastectomy radiotherapy on OS, DFS, and LRFS using Kaplan-Meier survival curves. These data indicated post-mastectomy radiotherapy did not impact OS, DFS, and LRFS in triple negative breast cancer patients with 1–3 positive nodes (*p* = 0.849, *p* = 0.860, and *p* = 0.162, respectively, Figure 3a, 3b, 3c).

**Figure 4:**
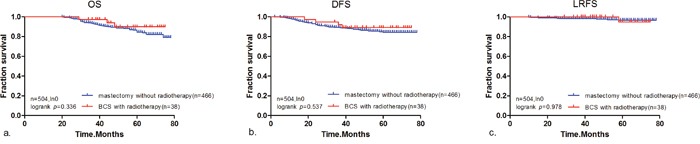
Kaplan-Meier analysis showing **(a)** OS, **(b)** DFS, and **(c)** LRFS for patients with T1/T2 triple negative breast cancer patients with no positive lymph nodes who were treated with breast-conserving surgery with post-operative radiotherapy (n = 38) or mastectomy alone (n = 466).

Of the 504 patients with negative nodes, 38 were treated with breast-conserving surgery and post-operative radiotherapy and 466 with mastectomy alone. The prognosis of triple negative breast cancer patients (OS, DFS, and LRFS) without lymph node metastasis who underwent breast-conserving surgery and post-operative radiotherapy was similar to that of patients who were treated with mastectomy alone (*p* = 0.336, *p* = 0.537, and *p* = 0.978, respectively, Figure 4a, 4b, 4c). Our results indicate that patients treated with breast-conserving surgery and post-operative radiotherapy achieved an equivalent prognosis to patients treated with mastectomy alone.

## DISCUSSION

Population-based studies have demonstrated that triple negative breast cancer patients have an increased risk of early metastasis and local recurrence compared to patients with other subtypes of breast cancer [23]. There are several conflicting reports regarding the value of post-mastectomy radiotherapy for the treatment of triple negative breast cancer. Data from the Early Breast Cancer Trialists’ Collaborative Group [10, 11] indicated that radiotherapy could improve survival. A prospective, randomized controlled multi-center trial in China also demonstrated that post-mastectomy radiotherapy significantly improved LRFS and OS in women with stage I and II triple negative breast cancer compared to mastectomy alone [18]. However, a Canadian study showed that breast-conserving surgery in combination with post-operative radiotherapy improved LRFS in patients with T1-2/N0 triple negative breast cancer compared to mastectomy alone, but did not significantly impact OS [12].

Data from the Danish Breast Cancer Cooperative Group 82b and 82c trials indicated post-mastectomy radiotherapy was not beneficial for triple negative breast cancer patients who had positive lymph nodes and/or T3-4 tumors [24]. However, these findings were not confirmed by other studies [19–22]. A population-based study from Sahlgrenska University Hospital reported that post-operative radiotherapy had no impact on the survival of patients from two similar regions of Sweden who were managed similarly. However, they did find that radiotherapy was indicated for patients with 1–3 positive nodes [25]. Finally, McBride et al. reported that patients with 1–3 positive nodes without risk features had a low rate of local recurrence, even in the absence of radiotherapy [16].

In this study, the primary outcome was relative survival. We analyzed differences in survival among triple negative breast cancer patients with either 1–3 or ≥ 4 positive nodes who were treated with similar surgical procedures and adjuvant chemotherapy with the exception of post-mastectomy radiotherapy All patients received adjuvant therapy. Therefore, the relative effects of minor differences in chemotherapeutics were not significant.

Post-mastectomy radiotherapy improved OS, DFS, and LRFS in triple negative breast cancer patients with ≥ 4 positive nodes. Cox regression analysis showed that post-mastectomy radiotherapy was also a significant prognostic factor. However, it did not improve survival in triple negative breast cancer patients with 1–3 positive nodes. The American Society of Clinical Oncology has recommended that patients with ≥ 4 positive nodes undergo routine post-mastectomy radiotherapy [26]. Post-mastectomy radiotherapy is undecided for patients with 1–3 positive nodes or with T1 or T2 tumors. Our results have potential to add new knowledge for these recommendations.

Bassam et al. reported that women with T1-2/N0 triple negative breast cancer who were treated with modified radical mastectomy without post-mastectomy radiotherapy had an increased risk of loco-regional recurrence compared to those treated with lumpectomy and adjuvant radiotherapy [26]. Decisions regarding the surgical approach for breast cancer treatment may reflect economic and cultural differences. Breast-conserving surgery is the primary surgical treatment for breast cancer worldwide. Approximately 60%–70% of patients with stage 0−II breast cancer in the United States undergo breast-conserving surgery [27]. However, a multicenter retrospective study in China indicated that modified radical mastectomy is still the most common surgical approach in China for breast cancer treatment [28].

Data from the Hong Kong Breast Cancer Registry indicated that there was a lower rate of breast-conserving surgery among Chinese patients with early invasive breast cancer [29]. A retrospective study that assessed the surgical management of breast cancer at a single center in Shanghai from 1999–2013 reported that 81.7% of the patients underwent mastectomy while only 15.2% underwent breast-conserving surgery. In addition, only 21.5% (606/2811) of ER-negative breast cancer patients underwent breast-conserving surgery. Only approximately 3.27% of triple negative breast cancer patients were treated with breast-conserving surgery [30]. The number of patients who underwent breast-conserving surgery was also low among T1/T2 triple negative breast cancer patients from the Heilongjiang Province in China. In our study, the prognosis of triple negative breast cancer patients who did not have lymph node metastasis and who were treated with breast-conserving surgery and post-operative radiotherapy was similar to that of patients treated with mastectomy alone. Because we had a limited number of patients who underwent breast-conserving surgery in our province, additional studies are needed to confirm our findings.

Our data suggest that post-mastectomy radiotherapy is beneficial in triple negative breast cancer patients with ≥ 4 positive nodes, but that there is little to no advantage of radiotherapy for patients with 1–3 positive nodes. These data are important given the well-known side effects of loco-regional, post-mastectomy radiotherapy [31–33]. Our findings have clear implications for the management of triple negative breast cancer patients with 1–3 or ≥ 4 positive lymph nodes. Additional studies with larger sample sizes are needed to confirm the importance of post-mastectomy radiotherapy for triple negative breast cancer patients with ≥ 4 positive lymph nodes.

## MATERIALS AND METHODS

All medical records were collected with the consent of the patients. The study protocol was approved by Harbin Medical University Cancer Hospital, Harbin, China. The inclusion criteria were (1) primary, operable triple negative breast cancer with tumor size ≤ 5 cm; and (2) clinical data available between the initial diagnosis and clinical follow-up. The exclusion criteria were (1) locally advanced disease with recurrent tumors, (2) treatment with neo-adjuvant chemotherapy, (3) metastatic disease, (4) tumor size > 5 cm, (5) the presence of too few cases (number<5) which were no advantage of statistical analysis.

The primary outcome of the study was mortality from all causes. Treatment data and patient clinical features were analyzed. Treatment data included the surgical approach (mastectomy or breast-conserving surgery), chemotherapy (yes, no), and adjuvant radiotherapy (yes, no). Surgical methods included modified radical mastectomy, mastectomy with sentinel lymph node biopsy, and breast-conserving surgery. Modified radical mastectomy and mastectomy with sentinel lymph node biopsy were collectively referred to as mastectomy. Tumor characteristics included the histological tumor size (in cm), number of positive lymph nodes, histological grade, Ki67 index, and P53 status.

ER, PR, and HER2 status was assessed by immunohistochemistry. We considered > 10% nuclear staining of ER and PR in the invasive component of the tumor to be positive. The intensity of the anti-HER2 staining was divided into four grades from 0–3 such that 0 was negative, 1 was slightly positive, 2 was indeterminate, and 3 was positive, as described previously [34, 35]. We re-categorized grades 0 and 1 as negative, and grade 3 was considered positive. Fluorescent *in situ* hybridization was performed on all grade 2 samples. Samples with a < 2-fold change in expression were considered negative, and samples with a > 2-fold change were considered positive for *HER2* gene amplification [36]. Tumor samples with Ki67 ≥ 14% were considered highly proliferative [37].

### Follow-up

All patients we analyzed were followed-up every 3–6 months for the first 5 years and every 12 months thereafter. The clinical and pathological records of all patients in the study were reviewed regularly as part of the analysis. Patients were followed for ≥ 5 years at the Harbin Medical University Cancer Hospital or until they died. We evaluated OS, DFS, and LRFS. Survival time was defined as the period from the date of diagnosis to either the date of death or the end of the study (June 31, 2015).

### Statistical analysis

All statistical analyses were performed using SPSS 17.0 for Windows. Survival curves were plotted using the Kaplan-Meier method and differences were assessed using log-rank tests. The influence of different variables on survival was assessed using the Cox univariate and multivariate regression analyses. *P* values <0.05 were considered statistically significant.

## SUPPLEMENTARY TABLES


