# Supplementary material for: Post-operative radiotherapy is beneficial for T1/T2 triple negative breast cancer patients with four or more positive lymph nodes
Source: Oncotarget. 2017 Apr 18;8(26):42917–25. doi: 10.18632/oncotarget.17170 (PMC5522115; doi:10.18632/oncotarget.17170)
Supplement: Supplementary file 1 [file oncotarget-08-42917-s001.pdf]

Post-operative radiotherapy is beneficial for T1/T2 triple negative breast cancer patients with four or more positive lymph nodes

SUPPLEMENTARY MATERIALS

SUPPLEMENTARY TABLES

Supplementary Table 1: Prognostic factors of DFS in triple negative breast cancer patients with ≥ 4 positive lymph nodes using the Cox proportional hazards model

Variables	Hazard Ratio (Univariate 95% CI)	*p*-value	Hazard Ratio (Multivariate 95% CI)	*p*-value
Age (years)				
≥45 vs. <45	1.36(0.829-2.231)	0.223		
Tumor size				
≥2cm vs. <2cm	2.106(1.142-3.386)	0.017	2.128(1.153-3.927)	0.016
Histological grade				
G1-2 vs. G3	0.797(0.462-1.377)	0.417		
Ki67 status				
≥14% vs.< 14%	1.012(0.639-1.602)	0.96		
P53 status				
Positive vs. Negative	1.234(0.795-1.915)	0.348		
Post-mastectomy radiotherapy				
Yes VS. No	0.633(0.412-0.974)	0.038	0.627(0.407-0.964)	0.034

Supplementary Table 2: Prognostic factors of LRFS in triple negative breast cancer patients with ≥ 4 positive lymph nodes using the Cox proportional hazards model

Variables	Hazard Ratio (Univariate 95% CI)	*p*-value	Hazard Ratio (Multivariate 95% CI)	*p*-value
Age (years)				
≥45 vs. <45	0.903(0.466-1.750)	0.763		
Tumor size				
≥2cm vs. <2cm	4.367(1.345-14.177)	0.014	4.484(1.381-14.562)	0.013
Histological grade				
G1-2 vs. G3	1.328(0.661-2.667)	0.426		
Ki67 status				
≥14% vs.< 14%	0.904(0.472-1.731)	0.76		
P53 status				
Positive vs. Negative	1.550(0.833-2.885)	0.166		
Post-mastectomy radiotherapy				
Yes VS. No	0.456(0.242-0.859)	0.015	0.445(0.236-0.837)	0.012
